# Evaluation of the Allplex GI Parasite and Helminth PCR Assay in a Belgian Travel Clinic

**DOI:** 10.3390/diagnostics14181998

**Published:** 2024-09-10

**Authors:** Jasmine Coppens, Charlotte Drieghe, Idzi Potters, Jean-Marc Schwob, Marjan Van Esbroeck

**Affiliations:** 1 Department of Clinical Sciences, Institute of Tropical Medicine, Nationalestraat 155, 2000 Antwerp, Belgium; 2 Division of Tropical and Humanitarian Medicine, Geneva University Hospitals, 1205 Geneva, Switzerland; 3 Bacteriology and Parasitology Laboratory, Geneva University Hospitals, 1205 Geneva, Switzerland

**Keywords:** protozoa, helminths, parasites, multiplex PCR, microscopy

## Abstract

Recently a number of broad-range stool parasite PCR assays have been developed. However, there is ongoing disagreement regarding their diagnostic performance, as various studies have produced contradictory results. In this study, we compared the diagnostic accuracy of the Seegene Allplex GI-Parasite and Allplex GI-Helminth assays (SA) with the conventional methods used at the travel clinic of the Institute of Tropical Medicine (ITM) including microscopy, antigen testing, and molecular detection in order to provide insights into the strengths and limitations of this diagnostic tool which may be crucial to select the most appropriate diagnostic tools for the suspected pathogen. A total of 97 native stool samples from 95 patients with suspected gastrointestinal illness were analyzed, including 26 from a frozen collection and 71 prospectively collected samples. The diagnostic performance of SA was notably superior to the conventional workflow in detecting *Dientamoeba fragilis* (sensitivity 100% vs. 47.4%) and *Blastocystis hominis* (sensitivity 95% vs. 77.5%). SA had a comparable performance with the conventional workflow in detecting pathogenic protozoa (sensitivity 90% vs. 95%). In contrast, SA had a much lower diagnostic performance in detecting helminths (59.1%) compared to the conventional workflow (100%). We conclude that the Seegene Allplex GI-Parasite assay may be useful for protozoa screening in low-endemic industrialized countries. However, the Allplex GI-Helminth assay is not recommended due to its suboptimal performance compared to microscopy.

## 1. Introduction

PCR has resulted in significant advancements in microbiological diagnostics by serving as the gold standard for diagnosing viral diseases [1]. Also, its superior speed and sensitivity make it particular valuable for identifying bacterial pathogens [1]. For these reasons, syndromic broad-range PCR panels have been increasingly used as first-line diagnostic tools for meningitis and severe acute diarrhea [2,3].

While PCR shows higher sensitivity than traditional methods in identifying protozoa, microscopic examination remains the gold standard for the diagnosis of most of the parasitic infections [1,4,5]. Although commercial testing may show higher efficiencies in detecting enteric pathogens [6,7], the effectiveness of molecular techniques in diagnosing helminthic infections is an ongoing discussion. Opinions differ between sources, with some claiming that their performances are below the standards set by traditional microscopic methods [8], while others argue that they perform comparably or even surpass them [9,10,11,12,13]. Molecular techniques have the disadvantage of being able to identify only a defined number of targets and requiring an expensive and complex infrastructure. In contrast, high-quality parasitological microscopic diagnosis requires human expertise, which is challenging to acquire outside reference centers due to the scarcity of positive samples and the time needed to become a well-trained parasite morphologist [4].

One of the commercially available platforms detecting helminths and protozoa in fecal samples is Seegene Allplex GI-Parasite and Allplex GI-Helminth (further referred to as SA), which detects six protozoa, and eight helminths and microsporidia, respectively. This platform integrates an automated DNA extraction and PCR setup device, STARlet (Seegene, Seoul, Republic of Korea), and a real-time PCR detection system CFX96 (Biorad, Hercules, CA, USA).

This study aimed to compare the diagnostic accuracy of the Seegene platform with that of the conventional workflow of the clinical laboratory of the Institute of Tropical Medicine (ITM). ITM, hosting a reference laboratory for parasitology, including microscopy, antigen testing and in-house PCR testing, examines samples from travelers and immigrants, with giardiasis and cryptosporidiosis being the leading protozoan infections in Belgium.

## 2. Methods

Study population: A total of 97 fecal samples from 95 patients with suspected gastrointestinal illness were included in the study. Eighty-one patients attended the ITM clinic and stool samples of the remaining 14 patients, attending other facilities, were sent to the clinical laboratory of the ITM in the scope of its reference function. Twenty-six stool samples from the period 2015 to 2023 were selected from a collection of fresh frozen positive samples stored at −80 °C and tested retrospectively, while 71 fresh samples were prospectively collected during the patients’ work-out between July and November 2023 on the condition that sufficient material was available to perform all tests.

Multiplex PCR procedure: The stool samples were processed according to the manufacturer’s instructions. About 1 g of each sample was suspended in 2 mL of eNAT medium. After vortex mixing, the suspensions were incubated for 10 min at room temperature. Subsequently, 1 mL of the suspension was transferred to a bead-beating tube, vortexed for 2 min, and stored at −20 °C until analysis. DNA extraction was performed using the Starlet extraction automate (Seegene), followed by PCR on the CFX96 (Biorad) cycler. A test result was considered positive in case of a well-defined exponential fluorescence curve that crossed the crossing threshold at a value of less than 45 for individual targets.

Diagnostic procedure at the clinical laboratory of the ITM (further referred to as the conventional method): tests on stool samples included microscopic examination of unstained and iodine-stained direct smears, wet mounts after formalin-ether concentration, iron hematoxylin Kinyoun staining of sodium acetate-acetic acid-formalin (SAF)-fixed stools, carbol-fuchsine staining on formalin-ether concentrates, the Baermann test method to detect *S. stercoralis* larvae, and copro-antigen enzyme-linked-immunosorbent-assays (ELISAs) for the detection of *Giardia*, *Cryptosporidium*, and *E. histolytica/E. dispar* (ProSpecT, OXOID Inc., Nepean, ON, Canada) depending on the request of the physician. Molecular methods included PCR to differentiate between *E. histolytica* and *E. dispar* performed on all samples positive for *E. histolytica/dispar* with microscopy or ELISA [14], PCR to detect *Strongyloides* (in case the conditions to perform the Baermann concentration were not met), *Enterocytozoon bieneusi*, and *Encephalitozoon* species on the request of the physician, and PCR to detect *G. duodenalis* (adapted from Verweij et al., 2003) [15], and *Cryptosporidium hominis* and *parvum* (adapted from Hadfield et al., 2011) used in case of discrepancy between microscopy and ELISA [16], and is this study additionally performed to confirm discrepancies between SA and the conventional method. Samples with weak positive PCR results (Ct value ≥ 38) were retested and considered negative if the second PCR result was negative. The detection of *Enterobius vermicularis* eggs was performed on demand using the scotch-tape method.

Detections of helminths and protozoa with the conventional method which were not found by SA were considered as true positive. Findings with SA and not with the conventional methods were confirmed by additional PCRs if available. Detections of *D. fragilis* and *B. hominis* with SA could not be confirmed with additional methods and results from SA were considered as true positives.

## 3. Results

Out of 97 stool samples, 63 tested positive for at least one parasite with SA, 60 were positive when using the conventional method, and 66 when considering a positive result with either method (Table 1).

Using the conventional workflow, 84 out of 104 (80.8%) protozoa were identified while SA found 75 (72.1%). The conventional workflow identified 26 protozoa that could not be detected with SA because they were not included in the panels, including 1 pathogenic species: *Cystoisospora belli*. SA outperformed the conventional workflow in detecting *D. fragilis* 19/19 (Sn 100%, CI 82.4–100%) vs. 9/19 (Sn 47.4%, CI 24.5–71.1%) and *B. hominis* 38/40 (Sn 95%, CI 83.1–99.4%) vs. 31/40 (Sn 77.5%, CI 61.6–89.2%). SA additionally detected one *G. duodenalis* (Ct value 38.24), one *Cryptosporidium* spp., and one *E. bieneusi*. The two last pathogens had not been looked for during the patient’s workout because the physician did not request the lab to do so but they were confirmed in retrospect by PCR. The *G. duodenalis* could not be confirmed with the conventional PCR but the sample was positive with an external PCR and the result was therefore considered truly positive. SA failed to identify one *E. histolytica* with a Ct value of 37.8 with the conventional PCR. The conventional workflow and SA correctly identified 19, respectively, 18 out of 20 (95% and 90%) pathogenic protozoa.

Concerning the helminths, only *Strongyloides* spp. (4/4) and *Hymenolepis* spp. (1/1) performed equally well with SA and the conventional workflow. SA detected a lower proportion of hookworms (2/3; 66.6%), *Ascaris* spp. (3/5; 60%*), Enterobius vermicularis* (2/3; 66.6%), and *Trichuris trichiura* (1/5; 20%) than the conventional method. No *Taenia* spp. was identified by either method, and *Schistosoma mansoni*, which is not included in SA panel, was found in one sample by the conventional workflow. SA correctly identified 13/22 (59.1%, CI 36.4–79.3%) pathogenic helminths compared to the conventional workflow which identified 22/22 (100%, CI 84.6–100%) (Table 2).

## 4. Discussion and Conclusions

For the detection of pathogenic protozoa, SA performed comparably to the conventional method used at ITM. This is in line with previous publications showing a high performance for the protozoa by SA [17,18]. Discrepancies between both methods, in the diagnosis of pathogenic protozoa, could be explained by a low concentration (high Ct value) of the protozoa (*E. histolytica* and *G. duodenalis*). The additional (*Cryptosporidium* and *E. bieneusi*) detected with SA were only in retrospect confirmed with the conventional PCR because the specific tests to detect these organisms were not requested by the physician and the detection of microsporidia is only reimbursed for immunocompromised patients in Belgium. The clinical significance of these pathogens can be questioned in these cases, as the low concentration is unlikely to correlate with symptomatic manifestations, and as diarrhea caused by *Cryptosporidium* spp. and microsporidia is self-limiting in immunocompetent patients. The detection of these pathogens with molecular methods could lead to expensive and unnecessary treatments. Most discrepancies among the identified protozoa concerned non-pathogenic species such as *E. coli* and *E. dispar*, and protozoa of controversial pathogenicity such as *D. fragilis* and *B. hominis* [19,20].

For the diagnosis of helminthic infections, SA performed equally well as the conventional method for *Strongyloides* spp. and *Hymenolepis* spp., although it did not allow to differentiate between *H. nana* and *H. diminuta*. However, SA demonstrated a lower sensitivity compared to microscopy for the detection of hookworms, *Ascaris lumbricoides*, *Enterobius vermicularis*, and *Trichuris trichiura*.

The performance of SA in the detection of *Taenia* spp. could not be evaluated because no samples containing *Taenia* spp. were included in the study. Helminth detection with SA was shown to be inferior, with only two out of eight targets performing as well as microscopy, resulting in a global sensitivity of around 60%. These results are consistent with other published research, which also showed insufficient helminths panel performance [8,21].

Potential reasons for the low sensitivity of SA versus microscopy are the fact that eggs are resistant to lysis, resulting in a limited release of DNA in the stool, and the inhomogeneous distribution of eggs and larvae in stools which can lead to sample errors. This emphasizes the need of appropriate pre-analytical steps. Another potential reason could be that part of the samples had been frozen before analysis and that SA was performed retrospectively, or because part of the samples had been frozen freshly, rather than in saline buffer [18,22]. However, in our experience and as reported by others, freezing the stools before molecular analysis rather tends to increase the sensitivity of parasite detection, especially for several helminths [14,23,24,25].

The advantage of multiplex PCR, and an argument used by companies to promote their use, is that they allow a syndromic testing approach. Consequently, it is difficult to understand why microsporidia have been included in the helminths and not in the protozoa panel, because microsporidia are associated with acute or no diarrhea at all and not with chronic diarrhea, except in immunocompromised patients. More generally, the composition of the so-called syndromic based panels seems strange as the panels cover pathogens corresponding to a broad spectrum of clinical symptoms from asymptomatic carriage and self-limiting diarrhea in immunocompetent individuals to potentially life-threatening conditions in immunocompromised patients. We feel that additional pathogens should have been included in the panel. Given the risk of neurocysticercosis depending on species, a distinction between *Taenia* species might have been useful. And the inclusion of more pathogenic species than *Hymenolepis* spp. in the panel, such as *Fasciola* spp. or Clonorchis/Opisthorchis, would have been relevant. Finally, *Schistosoma* spp., which affects 240 million people and is considered to have the third-highest global burden of neglected tropical diseases [26,27], should be included in a multiplex helminths panel.

A limitation of our study is the small sample size. Although all parasites present in the SA panel except *Taenia* spp., were included in the study, only a low number of samples positive for each species was analyzed. Another limitation of this study is the lack of specificity testing, as we did not confirm the presence of *D. fragilis* and *B. hominis* detected by SA.

In conclusion, the choice between SA and a conventional workflow may depend on the specific diagnostic priorities, considering the strengths and limitations observed in the detection of various parasites. The low sensitivity of the helminths panel of SA, not including the trematodes and several nematodes, raises the question of whether the method is accurate enough to be used in a travel setting. The suboptimal diagnostic performances compared to microscopy and the exclusion of several pathogens in the SA GI-Helminth assay advise caution against ruling out infections following negative results, particularly in migrants and travelers with a high pre-test probability. Seegene Allplex GI-Parasite may be useful for protozoa screening in low-endemic industrialized countries. Further research and validation may be necessary to refine the application of these methods in clinical practice.

## Figures and Tables

**Table 1:** Comparison of number of positive samples depending on the diagnostic methods.

	Allplex GI-Parasite and Allplex GI-Helminth Assays	Conventional Method	Both Seegene Allplex and Conventional Method
Negative	34	37	31
Positive	63	60	66

**Table 2:** Comparison of parasite detection by species and sensitivity depending on the diagnostic methods. Parasites in bold are included in the Seegene Allplex PCR panels (Seegene Allplex GI-Parasite = Protozoa, Seegene Allplex GI-Helminth = Helminths + Microsporidia). * *G. duodenalis* was confirmed by an additional external PCR. ITM: Institute of Tropical Medicine.

		Positive Samples at Institute of Tropical Medicine	Positive Samples with Seegene Allplex	Total Positive Samples	Sensitivity ITM Standard Workflow (%)	Sensitivity Seegene Allplex (%)
Protozoa	***Dientamoeba fragilis***	9	19	19	47.4%	100%
	***Blastocystis hominis***	31	38	40	77.5%	95%
	***Giardia duodenalis***	6	7 *	7	85.7%	100%
	***Entamoeba histolytica***	4	3	4	100%	75%
	***Cyclospora cayetanensis***	3	3	3	100%	100%
	***Cryptosporidium*** **spp.**	5	5	5	100%	100%
	*Cystoisospora belli*	1	0	1	100%	0%
	*Endolimax nana*	6	0	6	100%	0%
	*Entamoeba dispar*	6	0	6	100%	0%
	*Entamoeba coli*	8	0	8	100%	0%
	*Entamoeba hartmanni*	2	0	2	100%	0%
	*Iodamoeba bütschlii*	2	0	2	100%	0%
	*Chilomastix mesnili*	1	0	1	100%	0%
Fungi	***Enterocytozoon/*** ***Encephalocytozoon***	4	4	4	100%	100%
Helminths	**Hookworms**	3	2	3	100%	66.7%
	***Ascaris*** **spp.**	5	3	5	100%	60%
	***Enterobius vermicularis***	3	2	3	100%	66.7%
	***Strongyloides*** **spp.**	4	4	4	100%	100%
	***Trichuris trichiura***	5	1	5	100%	20%
	***Hymenolepis*** **spp.**	1	1	1	100%	100%
	***Taenia*** **spp.**	0	0	0	-	-
	*Schistosoma mansoni*	1	0	1	100%	0%

## Data Availability

All relevant data have been presented in the text. Raw data can be made available upon reasonable request.
